# Sleep Apnea, Obesity, and Diabetes — an Intertwined Trio

**DOI:** 10.1007/s11892-023-01510-6

**Published:** 2023-05-06

**Authors:** Soumya Kurnool, Karen C. McCowen, Nicole A. Bernstein, Atul Malhotra

**Affiliations:** UC San Diego Department of Medicine, 9500 Gilman Drive, UC San Diego, La Jolla, CA 92037 USA

**Keywords:** Sleep, Apnea, Obesity, Metabolic, Diabetes, Vascular

## Abstract

**Purpose of Review:**

To synthesize the existing literature regarding the complex interplay between sleep disturbance, obesity, and diabetes. The review emphasizes the three pillars of health being diet, exercise, and sleep, with the notion that if one is ignored, then the other two could suffer.

**Recent Findings:**

Sleep deprivation is associated with incident obesity, perhaps mediated by dysregulation in leptin and ghrelin — hormones important in regulation of appetite. Sleep apnea is very common particularly among obese people with type 2 diabetes mellitus. Treatment of sleep apnea has clear symptomatic benefits although its impact on long-term cardiometabolic health is less clear.

**Summary:**

Sleep disturbance may be an important modifiable risk for patients at risk of cardiometabolic disease. An assessment of sleep health may be an important component of the comprehensive care of patients with obesity and diabetes mellitus.

## Introduction

**Fig. 1:**
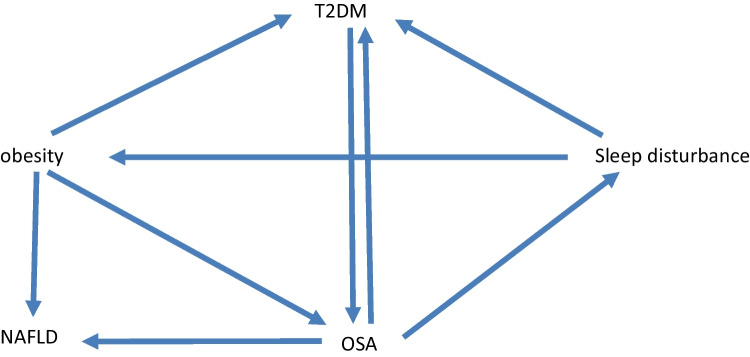
Proposed interactions between T2DM, obesity, OSA, and NAFLD. Obesity is a known risk factor for T2DM, OSA, and NAFLD. T2DM is linked bidirectionally with OSA with some evidence that each factor may worsen the disease process of the other. OSA may in theory directly impact NAFLD. There is some evidence that treatment of OSA itself may lead to weight gain likely due to behavioral changes

Obesity has been increasingly pervasive over the past several decades, in part due an intersection of genetic and behavioral factors, specifically increased consumption of high caloric processed foods, and more sedentary lifestyles [1–3]. Current estimates suggest that 1/3 of people in the USA have normal weight, 1/3 are overweight (defined by BMI 25–30 kg/m^2^), and 1/3 are obese (BMI > 30 kg/m^2^) [4]. Recent studies have suggested that based on the prevalence of obesity, life expectancy may start to decrease over time as a result [5]. The mechanisms underlying obesity related complications are complex, but type 2 diabetes mellitus (T2DM) and obstructive sleep apnea (OSA) are common comorbidities [6, 7]. Sleep deprivation is a common problem in today’s 24/7 society, with some evidence suggesting an important impact of sleep loss on overall health [8, 9]. OSA is estimated to affect almost 1 billion people worldwide [10, 11]. OSA is defined by repetitive collapse of the pharyngeal airway during sleep leading to derangements in gas exchange (hypoxemia and hypercapnia), as well as surges in catecholamines and other counter-regulatory hormones [12, 13]. Data from the SLEEP-AHEAD study (a sub-study under the LOOK-AHEAD obesity study) [6, 13] suggested that OSA (apnea hypopnea index — AHI>5.0/h) was present in 86.6% of obese patients with T2DM [7]. Of note, clinically important OSA (defined by AHI > 15/h) was present in 53.1% of this cohort. However, despite general acknowledgement that diet, exercise, and sleep are the three pillars of health [14, 15], most OSA remains undiagnosed and untreated [16, 17]. The interactions between obesity, OSA, and T2DM have been reviewed extensively and are summarized in Fig. 1. Although obesity is a major risk factor for OSA, as will be discussed further, treatment of OSA itself has been demonstrated in instances to lead to weight gain due to behavioral changes [18, 19]. Of note, OSA is characterized by sleep fragmentation whereas sleep deprivation refers to inadequate duration of sleep. The term sleep disturbance is used more generally to refer to various sleep pathologies including sleep fragmentation as well as sleep deprivation. We review here four specific topics in this context worthy of further consideration:

## Sleep Deprivation

1. Sleep deprivation. Sleep deprivation (defined by habitual sleep less than 7–9 h per night) has been clearly associated with incident obesity in many different epidemiological cohorts [20–22]. Patel et al. analyzed data from the Nurses’ Health Study and showed that women sleeping 5 h per night experienced more weight gain than those reporting 7 h of sleep per night [14]. Studies from the same cohort showed increased incident T2DM associated with short sleep as compared to people reporting adequate sleep duration [23]. Mechanistic studies have been performed by Spiegel et al. examining the impact of sleep deprivation on hormones that regulate appetite [24–27]. The authors randomized patients to obtain either inadequate sleep or adequate sleep during an in laboratory study in which constant glucose infusion was provided. They observed that sleep deprivation led to suppression of leptin levels and increase in ghrelin levels as compared to those sleeping adequately. Both hormones changed with sleep deprivation in a direction that would be predicted to stimulate appetite [26]. Thus, there is evidence that inadequate sleep may drive weight gain via dysregulation in satiety [28]. Of note, these hormonal changes have been somewhat inconsistent in various studies, amplifying the need for further research [29, 30]. However, despite reasonably compelling data regarding metabolic risk of sleep deprivation, OSA itself as a disease process may not increase body weight. In fact, several meta-analyses have suggested that treatment of OSA with CPAP (continuous positive airway pressure) is associated with weight gain, although the mechanisms are unclear [31–34].

## OSA and Type 2 Diabetes Mellitus

2. OSA and T2DM. Obesity is a common risk factor for both OSA and T2DM [35]. However, there are several proposed connections between OSA and T2DM independent of obesity. First, OSA may worsen T2DM and cause hyperglycemia with repetitive apnea inciting surges in catecholamines and other counter-regulatory hormones [36–38]. However, studies assessing the impact of OSA treatment with CPAP have shown mixed results without clear improvement of glycemic control [37, 39–41]. The reasons for this discrepancy are unclear but may relate to poor CPAP adherence, dietary indiscretion after symptomatic improvement with CPAP therapy, or patient selection in these studies. Second, T2DM may worsen OSA via neuromyopathic effects [38, 42]. Some data suggest that in patients with T2DM, OSA progresses in severity over time even without major weight gain [6, 43]. A theoretical explanation could be that T2DM is associated with abnormalities in control of breathing as well as neuromuscular effects impacting pharyngeal mechanics and worsening OSA. Third, T2DM and OSA may have synergistic effects on atherosclerotic and vascular disease risk. T2DM is known to impact the microcirculation and vascular smooth muscle, whereas OSA is thought to impact the endothelium preferentially [44, 45]. These findings support treatment of OSA especially in patients with T2DM to improve glycemic control and potentially reduce cardiometabolic risk. However, definitive randomized controlled trials regarding CPAP intervention to prevent cardiovascular disease are lacking [46, 47].
  Despite the numerous connections between OSA and T2DM, a major portion of patients with T2DM continue to be undertreated for OSA [48]. The Sleep-Ahead study was a sub-study under the Look-Ahead study which showed 86.6% of obese T2DM patients had clinically important OSA [13]. One year after the diagnosis was given to both patients and their physicians, > 95% of patients with OSA remained untreated [6, 7]. As a result, there is a need for increased awareness to promote OSA as a valuable therapeutic target with implications on cardiometabolic health for diabetic patients.

## OSA and Non-Alcoholic Fatty Liver Disease

3. OSA and non-alcoholic fatty liver disease (NAFLD): OSA and NAFLD are both associated with insulin resistance, although mechanistic research in this context is sparse. There are a number of studies showing a potential association between OSA and NAFLD [49]. NAFLD is sometimes regarded as a cardiovascular risk factor although the causal pathways are unclear and may well involve insulin resistance, obesity, and OSA at least to some extent [50]. A study by Corey et al. suggested that among patients undergoing bariatric surgery, the presence of OSA was a strong predictor of hepatic fibrosis [51]. In theory, OSA could contribute to NAFLD but may also increase risk of hepatic fibrosis and possibly hepatoma [52, 53]. A number of basic studies in mice have suggested the importance of intermittent hypoxia in contributing to liver abnormalities [54, 55]. Kallwitz et al. showed in obese patients with NAFLD that OSA was associated with elevated ALT levels and a possible link to histological evidence of liver disease [56]. These findings were supported by Mishra et al. who observed that OSA may be a risk factor for progression of NAFLD to NASH (non-alcoholic steatohepatitis) [57]. However, clinical data showing improvement in liver structure and function with OSA treatment are still evolving [58, 59].

## Treatment of OSA, Obesity, and T2DM

4. Treatment: The three pillars of health are often referred to as diet, exercise, and sleep [28]—with the dogma that if one ignores one, the other two will be compromised [20]. Thus, optimal health may require addressing all three factors [60]. However, sleep health is often ignored despite its potential contributions to treatment of OSA, obesity, and T2DM [61]. We focus on four treatments in this section:

- Exercise: Aerobic exercise is known to have benefits for overall health. Contracting muscle takes up glucose independently of the presence of insulin and potentiates insulin metabolic action after exercise, suggesting a major benefit to exercise in reducing hyperglycemia. Exercise has also been associated with improvement in daytime sleepiness and OSA severity even in the context of minimal weight change [62]. As a result, exercise training and rehabilitation may have a beneficial role as adjunctive treatment of OSA. Aerobic exercise is established as a vital component of weight management with additional benefit noted with high-intensity interval training, but likely works more effectively with concurrent diet and sleep interventions [60, 63–66].
- Weight loss medications: Weight loss medications developed over the past decade have become more efficacious in weight management and glycemic control. Previously, agents such as phentermine/topiramate, bupropion-naltrexone, and orlistat were commonly used and noted to have weight loss benefit, though use was limited by adverse effects, such as diarrhea with orlistat [67]. GLP1 (glucagon-like peptide 1) receptor agonists have emerged as promising treatments with daily liraglutide and weekly semaglutide injections which are FDA approved for both treatment of T2DM and obesity. Tirzepatide is a newer agent approved for T2DM therapy but with findings already suggestive of major weight loss in the diabetic population. However, this medication has not been adequately studied in nondiabetic patients at this time and is pending approval for use for obesity. The impact of these weight loss medications on OSA is less clear and is a topic of ongoing investigation (NCT05412004). One such study was the SCALE Sleep Apnea trial which evaluated the effects of liraglutide 3.0 mg daily in nondiabetic patients with diagnosed OSA but not on CPAP, which found a statistically significant reduction in AHI compared to placebo (− 12.2 events vs − 6.1 events, *p* = 0.0150), likely mediated through weight loss (− 5.7% compared to placebo group − 1.6%) [68]. Treatment of OSA and T2DM via weight loss medications may be a viable approach to minimize cardiometabolic risk [69–71].
- Bariatric surgery: Metabolic and bariatric surgery is increasingly used to facilitate major weight loss mostly via sleeve gastrectomy and Roux-en-Y bypass procedures which have shown the greatest efficacy. Over time, surgical techniques have evolved to help these processes become minimally invasive. Several studies confirm a durable weight loss following bariatric surgery as well as significant improvement (and sometimes resolution) of both T2DM and OSA. In some studies, OSA can recur over time, in part mediated by weight re-gain with aging and redistribution of body fat. Thus, long-term follow-up of these patients is recommended to optimize cardiometabolic risk following bariatric surgery [72–75].
- CPAP: Nasal CPAP has been shown to improve daytime symptoms of OSA, including sleepiness and daily functioning, and blood pressure [76, 77]. Although CPAP is frequently regarded as poorly tolerated, recent data suggest that adherence can be achieved in most patients given modern therapy, education, and troubleshooting of the mask interface. Patient engagement tools have been shown to achieve 87% adherence based on US Medicare criteria, suggesting that treatment of OSA can be achieved consistently with CPAP therapy [78]. However, there is general acknowledgement that alternative therapies will be required to optimize OSA treatment in addition to CPAP [79]. OSA is now recognized as having varying endophenotypes that need to be recognized rather than employing the current “one-size-fits-all” approach [80]. Despite suggestive observational data, definitive data from randomized trials are not available to show that CPAP leads to reductions in stroke or myocardial infarction [81]. As mentioned, data are equivocal regarding the benefits of CPAP related to glycemic control.
  Of note, CPAP has been associated with weight gain in some studies although the mechanisms are unclear. Some authors have measured extracellular fluid volume and have suggested that CPAP may cause fluid accumulation leading to weight gain [34]. Other authors have measured respiratory work associated with repetitive obstructive apnea and suggested that CPAP may reduce caloric expenditure by reducing the work of breathing in patients with OSA [82]. One concept that has been observed clinically is that some OSA patients when treated with CPAP have improvement in energy and quality of life and resume normal social activities leading to increased dietary indiscretion (i.e., eating out more often or having a beer with friends) and weight gain. Some hormonal theories have also been proposed regarding CPAP-induced weight gain, although the exact mechanism remains unclear [83].

## Conclusions

In summary, there are numerous interactions between obesity, OSA, and T2DM that impact cardiovascular and metabolic health. Sleep disturbance is an important and underrecognized risk factor which can be targeted via treatment of OSA or improving sleep hygiene to prevent progression of T2DM and obesity. Although treatment of OSA with CPAP does not clearly improve glycemic control, OSA remains an important therapeutic target for patients with T2DM to reduce cardiometabolic risk. OSA remains an underdiagnosed and undertreated condition in patients with T2DM, likely due to lack of awareness or systematic screening protocols. By integrating sleep health as a consideration in the treatment in all patients, there is potential for prevention of obesity, OSA, and T2DM and an improved quality of life.

## Declarations

### Human/Animal Studies

This article does not contain any studies with human or animal subjects performed by any of the authors.
